# ZBTB7A regulates LncRNA HOTAIR-mediated ELAVL1/SOX17 axis to inhibit malignancy and angiogenesis in endometrial carcinoma

**DOI:** 10.1007/s00432-024-05860-w

**Published:** 2024-07-09

**Authors:** Xiao-Hui Zhang, Shu-Wei Wu, Yi-Fan Feng, Yang-Qin Xie, Min Li, Ping Hu, Yunxia Cao

**Affiliations:** 1 Department of Obstetrics and Gynecology, The First Affiliated Hospital of Anhui Medical University, 218, Jixi Road, Hefei, Anhui Province 230022 P. R. China; 2 NHC Key Laboratory of Study on Abnormal Gametes and Reproductive Tract (Anhui Medical University), 218, Jixi Road, Hefei, Anhui Province 230032 P. R. China; 3 Key Laboratory of Population Health Across Life Cycle (Anhui Medical University), Ministry of Education of the People’s Republic of China, 218, Jixi Road, Hefei, Anhui Province 230032 P. R. China; 4 Anhui Province Key Laboratory of Reproductive Health and Genetics, 218, Jixi Road, Hefei, Anhui Province 230032 P. R. China; 5 Biopreservation and Artificial Organs, Anhui Provincial Engineering Research Center, Anhui Medical University, 218, Jixi Road, Hefei, Anhui Province 230032 P. R. China; 6 Department of Obstetrics and Gynecology, The Second Affiliated Hospital of Anhui Medical University, Hefei, Anhui Province 230022 P. R. China

**Keywords:** ZBTB7A, Endometrial carcinoma, HOTAIR, SOX17, Wnt-β-catenin pathway

## Abstract

**Background:**

Endometrial cancer (EC) is the sixth most frequent cancer in women worldwide and has higher fatality rates. The pathophysiology of EC is complex, and there are currently no reliable methods for diagnosing and treating the condition. Long non-coding RNA (lncRNA), according to mounting evidence, is vital to the pathophysiology of EC. HOTAIR is regarded as a significant prognostic indicator of EC. ZBTB7A decreased EC proliferation and migration, according to recent studies, however the underlying mechanism still needs to be clarified.

**Methods:**

The research utilized RT-qPCR to measure HOTAIR expression in clinical EC tissues and various EC cell lines. Kaplan-Meier survival analysis was employed to correlate HOTAIR levels with patient prognosis. Additionally, the study examined the interaction between ZBTB7A and HOTAIR using bioinformatics tools and ChIP assays. The experimental approach also involved manipulating the expression levels of HOTAIR and ZBTB7A in EC cell lines and assessing the impact on various cellular processes and gene expression.

**Results:**

The study found significantly higher levels of HOTAIR in EC tissues compared to adjacent normal tissues, with high HOTAIR expression correlating with poorer survival rates and advanced cancer characteristics. EC cell lines like HEC-1 A and KLE showed higher HOTAIR levels compared to normal cells. Knockdown of HOTAIR in these cell lines reduced proliferation, angiogenesis, and migration. ZBTB7A was found to be inversely correlated with HOTAIR, and its overexpression led to a decrease in HOTAIR levels and a reduction in malignant cell behaviors. The study also uncovered that HOTAIR interacts with ELAVL1 to regulate SOX17, which in turn activates the Wnt/β-catenin pathway, promoting malignant behaviors in EC cells.

**Conclusion:**

HOTAIR is a critical regulator in EC, contributing to tumor growth and poor prognosis. Its interaction with ZBTB7A and regulation of SOX17 via the Wnt/β-catenin pathway underlines its potential as a therapeutic target.

## Highlights

The expressions of HOTAIR are positively related to SOX17, and both are significantly overexpressed in endometrial carcinoma.

The transcription factor ZBTB7A inactivates the transcription of HOTAIR by binding to the promoter region of HOTAIR.

The study uncovers a novel mechanism where HOTAIR recruits ELAVL1 to regulate SOX17, subsequently activating the Wnt/β-catenin pathway.

## Introduction

Endometrial carcinoma (EC) is a frequent heterogeneous reproductive system illness that is characterized by metastasis induced by aberrant proliferation and angiogenesis. EC is the sixth most prevalent female cancer in the globe and the most frequent gynecological cancer in affluent nations (Vetter et al. 2020; Bray et al. 2018). There were 417,367 new confirmed cases worldwide in 2020, with a death rate of 24% (Sung et al. 2021). Although the treatment of early EC is relatively simple and usually successful with surgery, the treatment of late EC is difficult and the prognosis is poor, especially in the case of disease metastasis or recurrence, with a 5-year overall survival rate of only 15–17%, respectively(Green et al. 2020). EC is one of the few human malignant tumors that is becoming more lethal. Endometrial cancer has been increasing in frequency and death in recent years, posing a serious danger to female health (Gu et al. 2021). As a result, novel therapeutic targets and prognostic indicators to identify and treat this disease are urgently needed.

One of the POK family transcription factors known as ZBTB7A (Zinc Finger And BTB Domain Containing 7 A) is a multipotent transcription factor also known as POK (Pokemon), LRF (lymphoma-related factor), or FBI-1 (factor binding to IST protein 1) (Gupta et al. 2020). It can enlist several co-suppressors and binds specifically to short DNA recognition sites close to target genes (Cui et al. 2011; Choi et al. 2009; Jeon et al. 2008). Either enabling or disabling transcription, which is essential for cell division, proliferation, and other developmental phases (Gupta et al. 2020). Breast, prostate, and lung cancers, among others, have been linked to the genesis and spread of ZBTB7A overexpression (Singh et al. 2021; Chen et al. 2018; Aggarwal et al. 2011; Zhijun and Jingkang 2017). However, ZBTB7A down-regulation promotes tumor growth in some circumstances, suggesting that it may function as a tumor suppressor by preventing tumor metabolism and limiting glycolysis via transcription in human malignancies (Liu et al. 2014, 2016). ZBTB7A enhances P53 expression and triggers Caspase-dependent apoptosis through both mitochondrial and death receptor pathways in hepatocellular cancer (Zhang et al. 2013). ZBTB7A was identified to be underexpressed in EC by the GEPIA database, and we were able to predict the probable binding site of ZBTB7A to LncRNA HOTAIR using the JASPAR website. Additionally, earlier research has shown that ZBTB7A is dramatically down-regulated in EC and is crucial in controlling immune cell infiltration, which has diagnostic and prognostic relevance (Geng et al. 2020). There is no information available on the method through which ZBTB7A controls LncRNA HOTAIR in EC.

The HOTAIR gene, a long non-coding RNA (lncRNA), acts as a scaffold for histone modification complexes, notably through its 5’-terminal domain interacting with PRC2 (comprising EZH2, SUZ12, EED) and its 3’-terminal domain interacting with LSDI/COREST/REST complex. This interaction leads to modifications like methylation of H3K27 and H3K4me2. HOTAIR has been identified as a key player in various cancers, including breast cancer (Sørensen et al. 2013), hepatocellular carcinoma (Yang et al. 2011; Ishibashi et al. 2013), colorectal cancer (Kogo et al. 2011; Wu et al. 2014a, b), ovarian cancer (Li et al. 2015; Dai et al. 2021), and lung cancer (Ono et al. 2014; Liu et al. 2013). Its overexpression is associated with increased aggressiveness, metastasis, and poor prognosis in these cancers (Hajjari and Salavaty 2015). In various malignancies, HOTAIR is a predictor of overall survival and progression-free survival (Gupta et al. 2010a, b; Wu et al. 2014a, b; Hajjari et al. 2013; Endo et al. 2013). Specifically in endometrial cancer (EC), HOTAIR is found to be up-regulated and may serve as a diagnostic biomarker (He et al. 2014; Huang et al. 2014; Zhang et al. 2019). Further research is needed to fully understand its role in EC and its relationship with the transcription factor ZBTB7A.

SOX17 (SRY-box transcription factor 17) has been identified as a highly sensitive and specific marker for ovarian and endometrial carcinomas. It is particularly effective in diagnosing metastatic gynecologic carcinomas in cytology specimens, exhibiting high expression in all tested metastatic gynecologic carcinomas with strong nuclear expression. The remarkable sensitivity (100%) and specificity (98.2%) of SOX17 immunohistochemistry render it an invaluable asset for distinguishing metastatic gynecologic carcinomas in cytological samples(Satturwar et al. 2023). ELAVL1, an RNA-binding protein, plays a significant role in various cancers, including endometrial carcinoma. Its primary function involves regulating the stability and half-life of downstream mRNAs, which significantly affects cellular regulation. In cancer biology, ELAVL1 influences tumor proliferation, migration, and drug resistance. Its interaction with both microRNAs and long non-coding RNAs is crucial, as it can regulate their maturation or co-regulate with them to impact gene expression in the tumor microenvironment. This complex interaction between ELAVL1, RNA molecules, and miRNAs contributes to the progression and characteristics of endometrial carcinoma(Cai et al. 2022).

Based on our comprehensive analysis, we propose the following hypothesis: The transcription factor ZBTB7A suppresses the expression of ELAVL1 by transcriptionally inhibiting LncRNA HOTAIR, which in turn inhibits the malignant biological behavior and angiogenesis in endometrial carcinoma. This hypothesis has not yet been reported in the literature.

## Materials and methods

### Clinical sample collection

EC was extracted from 58 patients who underwent surgery in the First Affiliated Hospital of Anhui Medical University from May 2018 to July 2019 and stored at 80℃ in 58 cases of neighboring tissues removed at the junction between tumor and normal tissue. Preoperative chemical treatment or other malignancies were not present in these individuals with a comprehensive medical history. Meanwhile, patients were followed up every 6 months for 5 years after surgery. The Ethics Committee of the hospital approved the experimental agreement, all of the patients participating were given a permission form, and all of the formulae were supported by the Helsinki Declaration.

### Cell culture and transfection

The American Type Culture Collection provided EC cell lines (HEC-1 A, HEC-1B, RL95-2, KLE), human normal endometrial stromal cell lines (HESC cell line), and human umbilical vein endothelial cells (HUVEC) (ATCC; Manassas, VA, USA). HEC1A and HEC1B cells were grown in Mycos’5 A and MEM, respectively, both from Gibco in the USA. In DMEM/F12, KLE and RL95-2 were grown (Gibco). HESCs were cultured in Dulbecco’s Modified Eagle’s Medium (DMEM). The medium was supplemented with 10% Fetal bovine serum (FBS; Gibco) and 1% penicillin-streptomycin (Gibco). The cells were then incubated at 37 °C in a humidified environment with 5% CO_2_.

Wuhan Genesil Biotechnology designed and synthesised HOTAIR short hairpin RNA (shRNA) since it is highly expressed in EC tissue and cell lines, and sh-NC was used as a negative control in accordance with the suggested concentration guidelines (50 nM). GenePharma provided the over-expression plasmids oe-HOTAIR, oe-EZH2, and oe-ZBTB7A, and the empty oe-NC vector was used as the control. The transfection of the oe-NC, oe-HOTAIR, oe-SOX17, and sh-NC plasmids was carried out using Lipofectamine 2000 (Invitrogen, USA) in accordance with the manufacturer’s instructions on selected HEC-1 A and KLE cells. Co-transfection of oe-NC + oe- ZBTB7A and oe-ZBTB7A + oe-SOX17 into cells. Following transfection, total RNA and protein were extracted at 24 and 48 h, respectively.

### RNA extraction and RT-qPCR analysis

To isolate RNA, HEC-1 A and KLE cells were treated and transfected using the techniques described above. Using the TRIzol reagent (Invitrogen, Carlsbad, CA, USA), total RNA was isolated following the guidelines provided by the manufacturer. Subsequently, this RNA underwent reverse transcription into cDNA utilizing the PrimeScript RT reagent Kit (TaKaRa, Dalian, China), again adhering to the instructions specified by the manufacturer. Takara Bio Group’s SYBR Premix Ex Taq was used for the amplification reactions to determine SYBR®Green I expression levels, with a final reaction volume of 10 uL following the manufacturer’s recommendations. In qPCR detection, GAPDH was employed as the internal reference for HOTAIR, ZBTB7A. The ABI PRISM 7500 Sequence Detection System was used for the qPCR experiments (Applied Biosystems). The 2^−∆∆Ct^ method technique was used to determine the relative expression levels of HOTAIR, ZBTB7A mRNA. The primers are depicted below Table 1.

**Table 1:** primers

Gene	Forward primer (5’ to 3’)	Reverse primer (5’ to 3’)
HOTAIR	GGTAGAAAAAGCAACCACGAAGC	ACATAAACCTCTGTCTGTGAGTGCC
ZBTB7A	TGCAAGGTCCGCTTCACCAG	TGCAAGGTCCGCTTCACCAG
SOX17	AACGCCGAGTTGAGCAAGAT	CTTAAGAAAGGACGTGGCCG
GAPDH	CGGATTTGGTCGTATTGGG	CTGGAAGATGGTGATGGGATT

### Actinomycin D treatment

HEC-1 A and KLE cells were treated with 5 µg/mL actinomycin D (Sigma-Aldrich, USA) to inhibit transcription. Cells were harvested at 0, 4, 8, 12, and 24 h post-treatment. Total RNA was extracted at each time point using the RNeasy Mini Kit (Qiagen, Germany).

### Western blot analysis

RIPA (Sigma, USA) lysis buffer with a protease and phosphatase inhibitor cocktail was used to extract the protein from the cells (MCE, USA). Proteins from the extraction process were heated in loading buffer for 5 min at 95 °C, and then they were separated on a 10% SDS-PAGE gel. The polyvinylidene difluoride (PVDF) (Millipore, USA) membranes were incubated with primary antibodies for an overnight response at 4 °C after being transferred with proteins and blocked with 5% bovine serum albumin at room temperature for 1 h (dilution at 1:1000), Cell Signaling Technology (Danvers, Massachusetts, USA) provided the VEGFA, CD31, p-PI3K, and GAPDH; Abcam (Cambridge, Massachusetts) provided the SOX17, ELAVL1, Wnt-1, and β-catenin. The membranes underwent three washes with TBST containing 0.1% Tween 20, each for 10 min. Following this, they were incubated with secondary antibodies for one hour at ambient temperature. The ImageQuant LAS 4000 (GE Healthcare Life Sciences, Piscataway, NJ, USA) was used to capture images of all the bands, with GAPDH serving as the internal reference. The program ImageJ was used to evaluate the bans (version 1.44, National Institutes of Health, Bethesda, MD, USA).

### Colony formation assay

The cell suspensions were plated onto 6-well plates at the appropriate cell density based on their ability to proliferate. After 14 days of incubation with 5% CO_2_ at 37 °C and saturated humidity, the liquid can be changed after 7 days. Cells were fixed with PFA for 30 min before staining with 0.1% crystal violet for 30 min. The plates were then inverted and overlaid with a grid clear film. The rates of clone creation with more than 10 cells were determined.

### Tube formation in vitro assay

HUVECs were cultured in DMEM supplemented with 10% FBS, 1% penicillin-streptomycin solution, and a 5% CO2 incubator at 37 °C. HUVECs were added to the pre-cooled 96-well plate in 50 µL of growth factor-reduced matrix adhesive Matrigel. The 96-well plate was treated with the cells (1–2 × 10^4^). Centrifugation transfected HEC-1 A and KLE cells supernatant was added to each well to replace the centrifugally collected conditional culture media. In order to see and count the quantity of vascular endothelial cell tubules, the cells were placed in an incubator for standard culture for 24 h before being randomly picked by 5 fields and examined using Chemi Imager 5500 V2.03 software (Alpha Innotech, San Leandro, CA, USA).

### Transwell assay

Cells were cultured to 70–80% confluency, trypsinized, and suspended in serum-free medium. The invasion assay involved a Matrigel-coated upper compartment of a Transwell insert. Cells, at a density of 1 × 10^5 cells/well, were seeded in the upper chamber using a serum-free medium, while the lower chamber was filled with a medium containing 10% FBS, serving as a chemoattractant. After incubation at 37 °C in 5% CO_2_ for 24–48 h, non-migrated or non-invaded cells were removed. Cells that migrated or invaded to the lower surface of the membrane were immobilized, dyed using crystal violet, and subsequently quantified using a microscope. This was repeated across different groups to assess how genetic changes affect cell migration and invasion, providing insights into cancer metastasis.

### Dual-luciferase reporter assay

ZBTB7A binding sites on E3 of the HOTAIR promoter region were identified using an RNA-binding protein immunoprecipitation test, and the E3 wild and mutant binding sites were designed. E3-wild type (WT) /E3-mutant type (MUT) luciferase reporter plasmids were introduced into the polyclonal site (MCS) downstream of the luciferase gene in the double pmirGLO luciferase vector (Promega, Madison, USA). After co-transfection of the ZBTB7A construct or empty vector with pmirGLO reporter plasmids into cells, changes in luciferase activity were measured to determine if ZBTB7A operated on possible target genes.

### RNA pull-down assay

RNA pull-down experiments were used to identify HEC-1 A and KLE cells. GenePharma Company (Shanghai, China) transcribed HOTAIR in vitro and tagged it with biotin, while IgG served as a negative control. By following the guidelines of the Pierce Magnetic RNA-protein Pull-Down Kit (Thermo Fisher Scientific, Waltham, MA, USA), biotin-labeled RNA binding beads were prepared through incubation in a protein-RNA binding solution. Lysates were incubated with probe-RNA binding beads overnight at 4 ℃. The Western Blot experiment was used to see if certain RNA binding proteins interacted with RNA.

### Animal experiments

Animal studies were conducted out with the agreement of the First Affiliated Hospital of Anhui Medical University’s biomedical ethics committee. We created a tumour metastatic mode as well as a xenograft model to better understand the role of ZBTB7A in EC development, metastasis, and angiogenesis in vivo. Beijing Vital River Laboratory Animal Technology Co., Ltd. purchased female BALB/c nude mice (4–5 weeks old). In the xenograft model, mice were randomly assigned to one of two groups: oe-ZBTB7A (*n* = 6) and oe-NC (*n* = 6). HEC-1 A and KLE cells (5 × 10^7^ cells) transfected with oe-ZBTB7A or oe-NC were implanted subcutaneously into mice, respectively. Tumor volumes were measured every week after injection, and tumour weight was computed after 30 days using the formula (length width2).

### Tumour metastatic mode and HE staning

In order to create a tumour metastasis model, HEC-1 A and KLE cells transfected with oe-ZBTB7A or oe-NC were injected into 4-week-old nude mice through the tail vein (six in the experiment group and six in the control group), and lung tissue was examined for metastasis. Thirty days following injection, the number and size of metastases were counted. The pathological samples of the pulmonary nodules were collected, formalin-fixed, and cut into paraffin sections before being stained with H&E for histological analysis.

### Immunohistochemistry

The specimens were first fixed with 10% formalin, then traditional paraffin-embedded stone paraffin slices were submerged in turpentine and gradient alcohol for 10 min at room temperature, followed by an infusion with 3.0% H2O2, and finally cultured with citric acid buffer. VEFDA, Ki-67, and CD31 antibodies were used to identify and bind antigens in tissues. Restaining the cells and displaying the cell contour were accomplished using a second antibody detection with the chromogenic agent. The cells were stained, examined under a microscope, and taken photos of.

### Statistical analysis

GraphPad Prism 8 software (GraphPad Software Inc., La Jolla, California, USA) was used to analyse all data summaries, and Image-Pro Plus 6.0 software was used to process all images (Media Cybernetics, Silver Spring, MD, USA). Cross-group comparisons were carried out using bidirectional analysis of variance (ANOVA), followed by Tukey post-hoc testing. For survival and correlation analyses, the log-rank test and Pearson correlation coefficient were employed, respectively. The different groupings that were judged statistically significant were thought to be *P*<0.05 or *P*>0.01.

## Results

### HOTAIR was increased and correlated with patient prognosis in EC tissues and cell lines

**Fig. 1:**
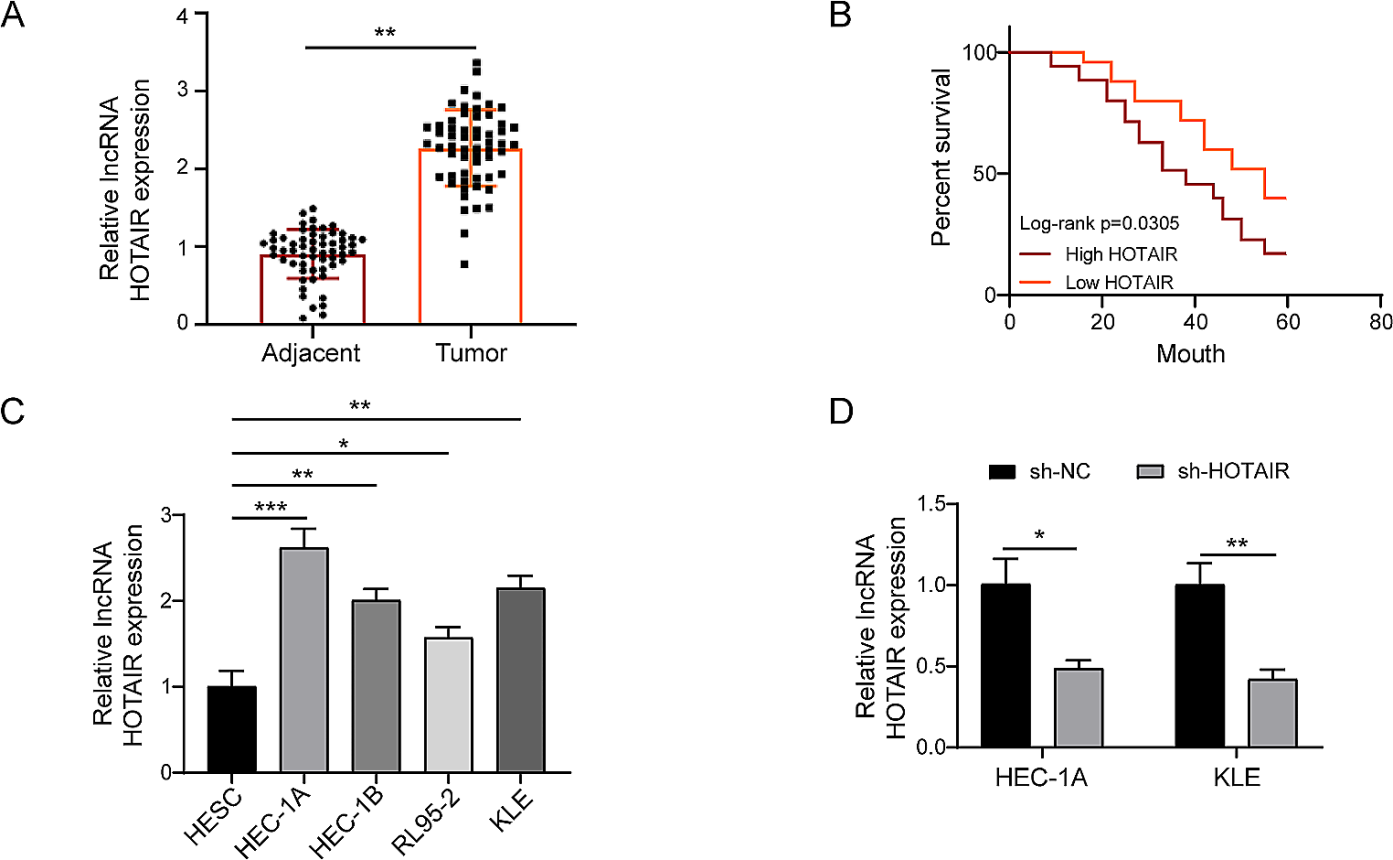
HOTAIR, a LncRNA, is increased in EC tissues and cell lines (**A**) RT-qPCR revealed that LncRNA HOTAIR expression was considerably greater in EC tissues (*n* = 58) than in neighbouring tissues (*n* = 58). (**B**) Kaplan-Meier survival curve analysis shows that EC tissues expressing high levels of HOTAIR had a considerably higher 5-year survival rate than neighbouring tissues expressing low levels of HOTAIR. (**C**) RT-qPCR study of HOTAIR expression levels in HEC-1 A, HEC-1B, RL95-2, and KLE cell lines. (**D**) HOTAIR knockdown drastically reduced HEC-1 A and KLE cell viability. Data from at least three replicates were presented as mean ± standard deviation (SD). *P < 0.05, **P < 0.01

Previous studies found that higher HOTAIR was associated with a worse outcome (He et al. 2014; Nyen et al. 2018). To further verify the oncogenic role of LNCRNAand choose an appropriate cell line to investigate the malignant biological activity of EC. The RT-qPCR technique was used to evaluate the expression level of the lncRNA HOTAIR in clinical sample tissues from patients with EC. We discovered that HOTAIR expression in EC tissues (*n* = 58) was considerably greater than that in neighbouring normal EC (*n* = 58) (Fig. 1A). To further understand the association between HOTAIR and patient prognosis, the Kaplan-Meier survival analysis was employed, which revealed that high HOTAIR expression was substantially connected with shorter survival in the 5-year survival rate of patients with EC (Fig. 1B).The LncRNA HOTAIR low expression group (*n* = 23) and the LncRNA HOTAIR high expression group (*n* = 35) were investigated in 58 patients with EC. According to the results, the expression of LncRNA HOTAIR was independent of age and histological grade, but high levels of expression were associated with FIGO stage, tumor size, muscular infiltration, and lymph node metastases. (Table 2). Furthermore, HOTAIR expression was considerably higher in EC cell lines such as HEC-1A, HEC-1B, RL95-2, and KLE compared to HESC (Fig. 1C), particularly in HEC-1A and KLE cells. We chose the cell lines with the greatest expression to create the sh-HOTAIR expression vector and transfected sh-NC or sh-HOTAIR plasmids into HEC-1A and KLE cells, respectively. RT-qPCR transfection efficiency study revealed that sh-HOTAIR had a greater knockdown effectiveness (Fig. 1D) and may be employed in future research. In conclusion, these findings suggest that HOTAIR was persistent and substantially expressed, and that it is strongly linked to tumour growth and poor prognosis in EC.

**Table 2:** The association between LncRNA HOTAIR expression and clinicopathologic features of EC patients

Clinicopathologic features	n = 58	LncRNA HOTAIR expression		*P*-value
		High (n = 35)	Low (n = 23)	
*Age (years)*				
< 50	20	12	8	0.2662
≥ 50	38	23	15	
*FIGO stage*				
I + II	32	15	17	0.0129^*^
III + IV	26	20	6	
*Tumor size*				
≤ 3cm	22	11	11	0.0374^*^
> 3cm	36	24	12	
*Muscular infiltration*				
< 1/2	23	9	14	0.0258^*^
≥ 1/2	35	26	9	
*Lymph node metastasis*				
No	34	18	16	0.0183^*^
Yes	24	17	7	
*Histological grade*				
Well	31	19	12	0.2952
Moderately/poorly	27	16	11	

^*^P < 0.05 indicates a statistically significant difference

### Reduced HOTAIR affected the proliferation, angiogenesis, and migration of EC cell lines

**Fig. 2:**
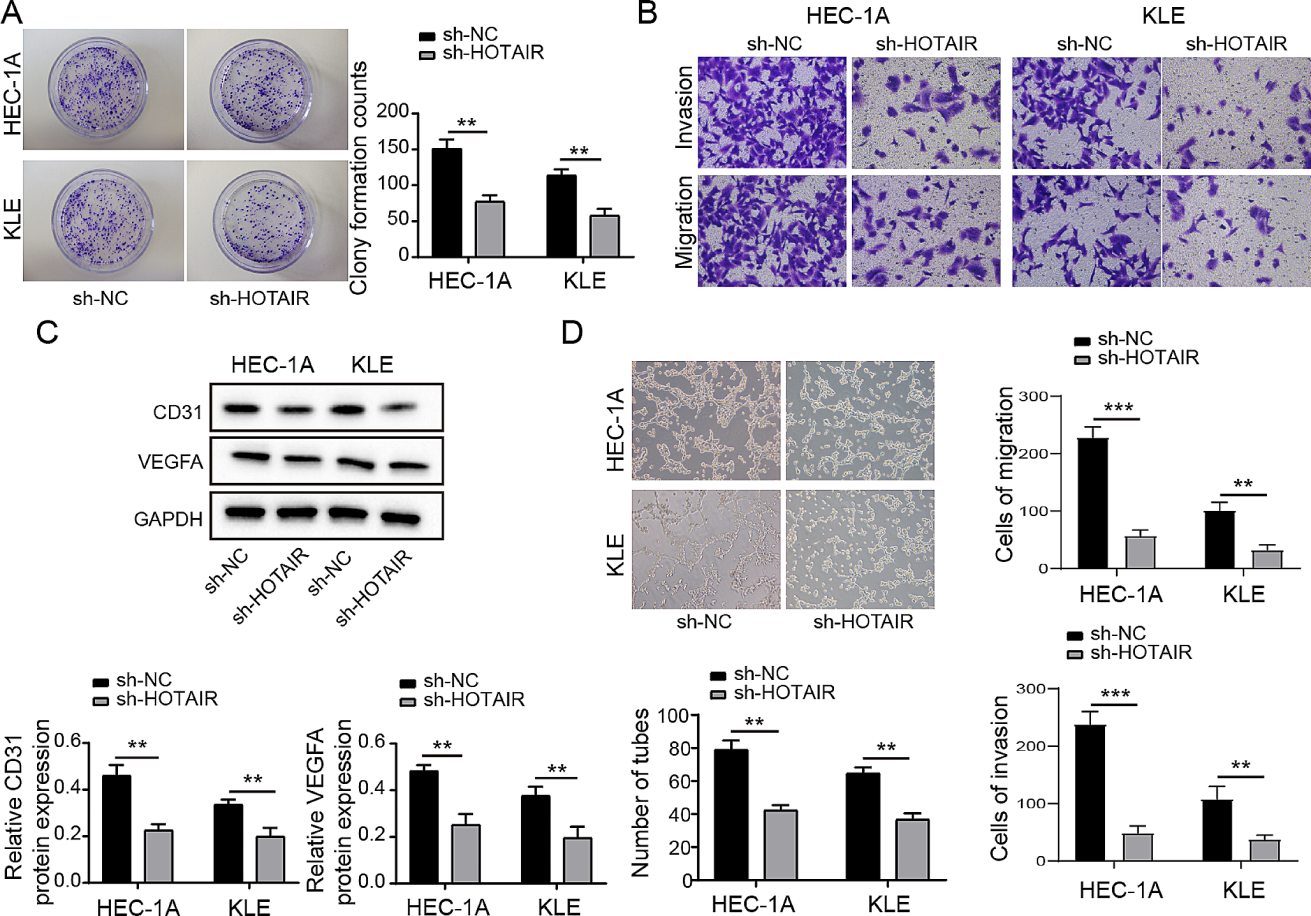
Knockdown of HOTAIR Inhibits Growth and Angiogenesis in Endometrial Cancer Cells, and Promotes Migration (**A**) The colony formation assay to measure HEC-1 A and KLE cells proliferation. (**B**) Transwell assay to assess cell invasion and migration. (**C**) Western Blot to measure the expression levels of CD31 and VEGFA in cells. (**D**) Blood vessels of HUVECs in the tumor-conditioned media from transfected HEC-1 A and KLE cells in the tube formation experiment. Data from at least three replicates were presented as mean ± standard deviation (SD). *P < 0.05, **P < 0.01

Our findings showed that HOTAIR fatigue has a strong negative influence on cell formation number using the colony formation experiment (Fig. 2A). knockdown of HOTAIR leads to a significant reduction in cell invasion and migration (Fig. 2B). By using Western blotting to measure the levels of angiogenesis-related proteins CD31 and VEGFA, it was discovered that the levels of these proteins were much lower in the sh-HOTAIR transfected group than in the sh-NC group (Fig. 2C). This suggested that HOTAIR and EC angiogenesis could be related. HUVECs were co-incubated with supernatants of transfected HEC-1 A and KLE cells for 12 h, and we saw that the capacity of these cells to form tubes was considerably decreased in the sh-HOTAIR group compared to the control sh-NC transfection group (Fig. 2D). According to all available information, HOTAIR is a critical regulator of EC cell proliferation, angiogenesis, and migration.

### The relationship between ZBTB7A and HOTAIR

**Fig. 3:**
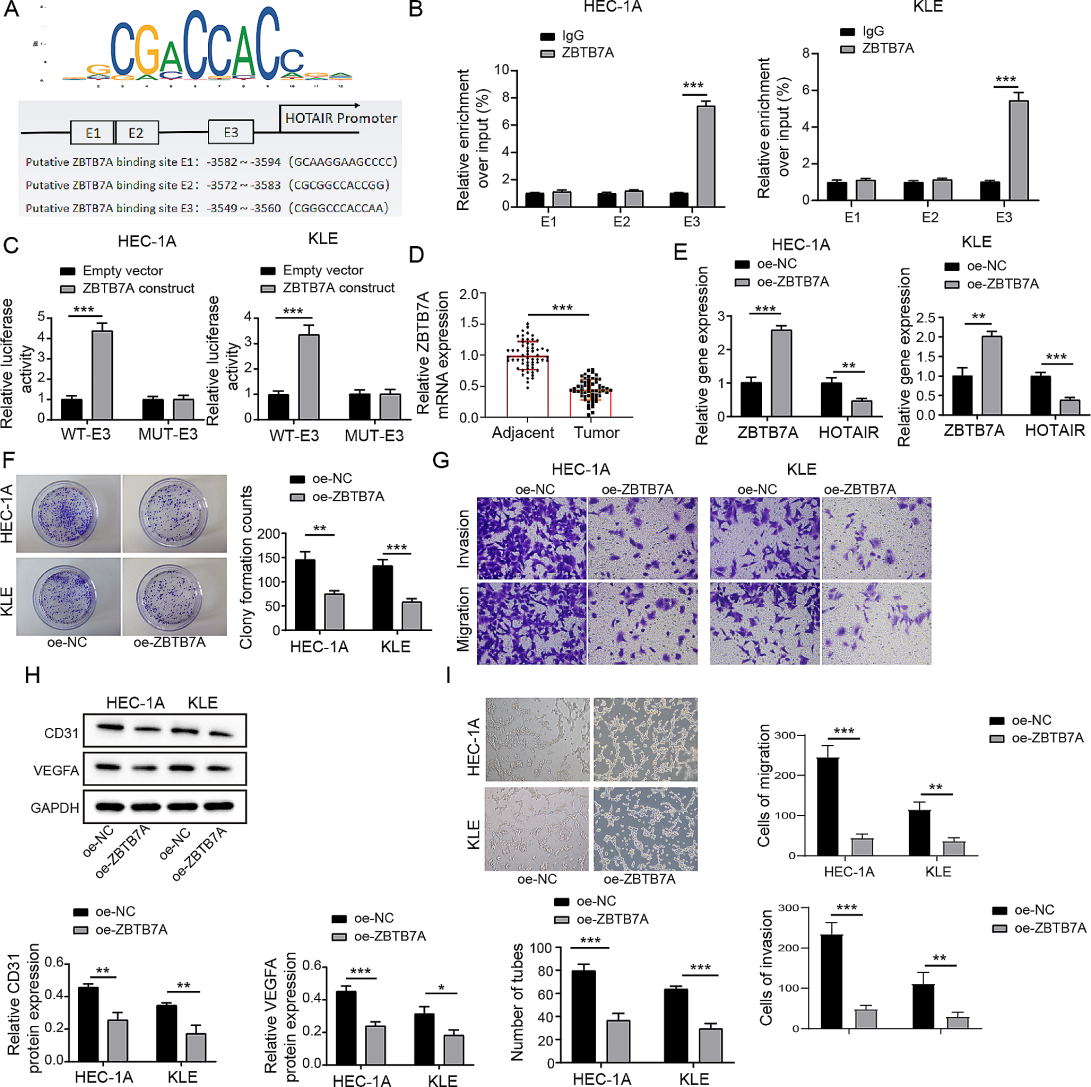
ZBTB7A Inhibits the Expression of HOTAIR by Binding to its Promoter Region, Thereby Suppressing Malignant Biological Behavior and Angiogenesis in Endometrial Cancer (**A**)The JASPAR website predicted the binding location and the first three binding sequences of ZBTB7A to the HOTAIR promoter region. In HEC-1 A and KLE cells, ChIP to reveal the binding location of ZBTB7A in the HOTAIR promoter region. (**C**) Dual-luciferase reporter experiment to demonstrat the effect of the ZBTB7A construct on the luciferase activity of the WT or MUT luciferase reporter plasmid. (**D**) qPCR to measure the expression levels of ZBTB7A in endometrial cancer tissues and adjacent non-cancerous tissues. (**E**) qPCR to detect the expression levels of ZBTB7A and HOTAIR in each cell group. (**F**) Plate cloning experiment to assess the number of cell clones formed. (**G**) Transwell assay to evaluate cell invasion and migration. (**H**) Western Blot to measure the expression levels of CD31 and VEGFA in cells. (**I**) Tube formation assay to quantify the number of blood vessels formed in. Data from at least three replicates were presented as mean ± standard deviation (SD). *P < 0.05, **P < 0.01

More and more evidence points to the role of non-coding RNAs and ZBTB7A malfunction in the growth and metastasis of human malignancies (Singh et al. 2021; Hao et al. 2020). . However, it is unknown if it can modulate HOTAIR transcription. We did bioinformatics research utilising the online tool JASPAR website (http://jaspar.genereg.net/) to further analyse whether ZBTB7A interacted with LncRNA HOTAIR. We retrieved the binding sites and the first three binding sequences of ZBTB7A in the HOTAIR promoter region from the website (Fig. 3A). Furthermore, we discovered that ZBTB7A had a lower EC in the GEPIA database (Tang et al. 2017). . The ChIP assay discovered that the binding site of ZBTB7A in the HOTAIR promoter region is E3, confirming the validity of the anticipated location, which is depicted in Fig. 3B.

The findings of the dual-luciferase test also revealed that luciferase activity decreased with the mutation of the E3 segment in the HOTAIR promoter region (Fig. 3C). Then, using RT-qPCR, we discovered that the expression level of ZBTB7A in EC tissues (*n* = 58) was considerably lower than that in nearby tissues (*n* = 58) (Fig. 3D). ZBTB7A was decreased in EC, as expected by bioinformatics.

Furthermore, RT-qPCR analysis revealed that ZBTB7A overexpression reduced the amount of HOTAIR in HEC-1 A and KLE cells transfected with oe-NC or oe-ZBTB7A (Fig. 3E). Meanwhile, the capacity of cells to proliferate after ZBTB7A overexpression was dramatically decreased when compared to the oe-NC group (Fig. 3F). The study noted a significant reduction in cell migration and invasion in HEC-1 A and KLE cell lines due to the overexpression of ZBTB7A, as illustrated in Fig. 3G. Likewise, using western blot analysis, there was a noticeable decrease in the protein expression levels of CD31 and VEGFA, depicted in Fig. 3H. Following that, a tube formation experiment revealed that ZBTB7A enrichment greatly reduces angiogenesis (Fig. 3I). According to the evidence presented above, the unusually low expression of ZBTB7A in EC may be one of the causes of the high expression of HOTAIR, boosting the malignant biological behaviour of EC.

### HOTAIR recruits ELAVL1 to regulate SOX17

**Fig. 4:**
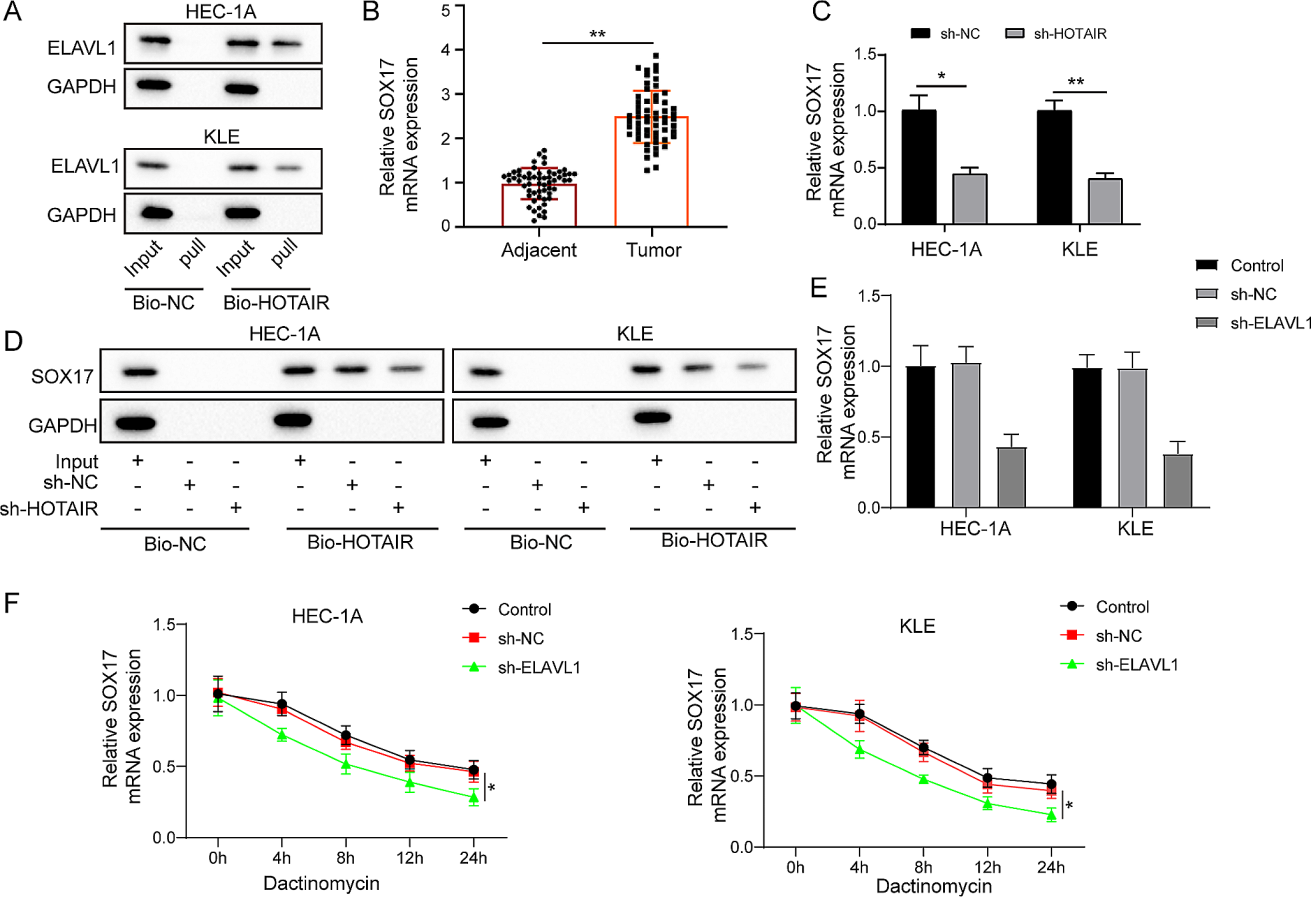
HOTAIR Recruits ELAVL1 to Regulate SOX17. (**A**) RNA pull-down and Western Blot analysis confirmed the interaction between HOTAIR and ELAVL1, with biotin-labeled HOTAIR probes successfully pulling down ELAVL1 protein. (**B**) qPCR to detect the expression level of SOX17 in endometrial cancer tissues. (**C**) qPCR to detect the level of SOX17. (**D**) RNA pull-down and WB analysis showed that in samples where the HOTAIR gene was knocked out (sh-HOTAIR), the level of SOX17 was significantly reduced compared to the control group (sh-NC). (**E**) qPCR to detect the level of SOX17. (**F**) Actinomycin D to examin the stability of SOX17 mRNA under sh-ELAVL1 treatment. Data from at least three replicates were presented as mean ± standard deviation (SD). *P < 0.05, **P < 0.01

Subsequently, we investigated the mechanism by which HOTAIR functions in EC. Through RNA pull-down and Western Blot (WB) analysis, we established a direct interaction between HOTAIR and ELAVL1. Experiments using biotin-labeled HOTAIR probes successfully pulled down and detected ELAVL1 protein, confirming the existence of this interaction (Fig. 4A). Quantitative PCR (qPCR) analysis revealed that the expression level of SOX17 in endometrial cancer tissues was significantly higher than in adjacent non-cancerous tissues. This finding suggests that SOX17 may play an important role in the development of endometrial cancer (Fig. 4B). After knockdown of HOTAIR, we observed a significant decrease in SOX17 expression levels. This indicates that HOTAIR may affect the biological characteristics of endometrial cancer by regulating the expression of SOX17 (Fig. 4C). Further RNA pull-down and WB analysis showed that in samples where the HOTAIR gene was knocked out (sh-HOTAIR), the level of SOX17 was significantly reduced compared to the control group (sh-NC). This result reinforces the crucial role of HOTAIR in regulating the expression of SOX17 (Fig. 4D). To further validate the role of ELAVL1 in the regulation of SOX17, we performed additional qPCR analysis to measure SOX17 RNA expression after ELAVL1 knockdown. The results showed a significant reduction in SOX17 mRNA levels in both HEC-1 A and KLE cell lines when ELAVL1 was knocked down (Fig. 4E). Moreover, actinomycin D chase assays were conducted to evaluate the impact of ELAVL1 on SOX17 mRNA stability. The findings revealed that knockdown of ELAVL1 markedly decreased the stability of SOX17 mRNA in HEC-1 A and KLE cell lines (Fig. 4F). In summary, our study reveals that HOTAIR recruits ELAVL1 to regulate SOX17.

### SOX17 promotes malignant biological behavior and angiogenesis in endometrial cancer cells by activating the Wnt/β-catenin pathway

**Fig. 5:**
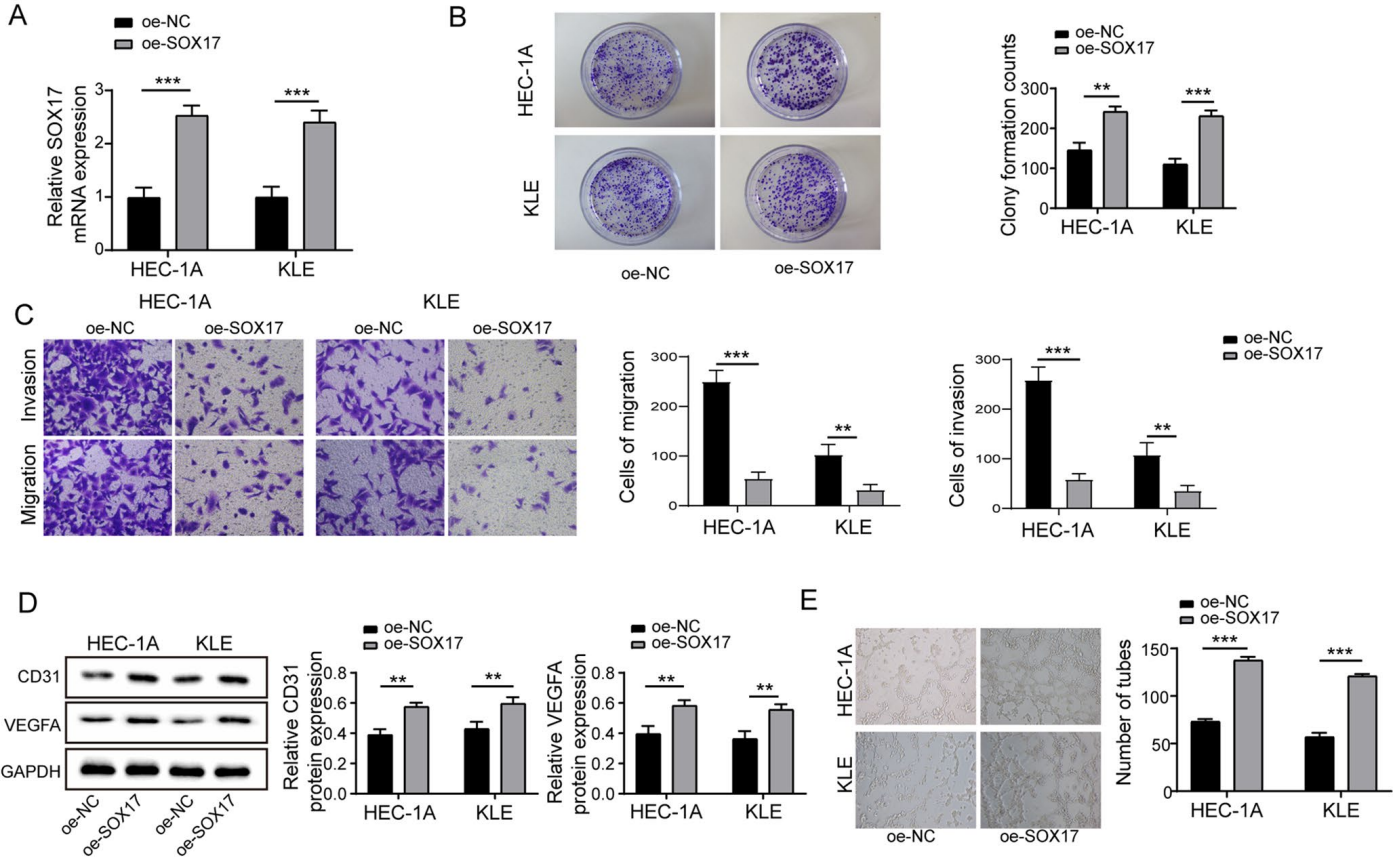
SOX17 Promotes Malignant Biological Behavior and Angiogenesis in Endometrial Cancer Cells by Activating the Wnt/β-Catenin Pathway (**A**) After overexpression of SOX17, the levels of SOX17 were significantly increased. (**B**) Plate cloning experiments demonstrated that cells with overexpressed SOX17 exhibited a significantly enhanced proliferative capacity. (**C**) Transwell assays showed increased cell invasion and migration following SOX17 overexpression. (**D**) Western Blot analysis indicated that the protein expression levels of CD31 and VEGFA significantly increased after SOX17 overexpression. (**E**) Tube formation assays revealed a significant increase in the number of blood vessels formed in cells overexpressing SOX17. Data from at least three replicates were presented as mean ± standard deviation (SD). *P < 0.05, **P < 0.01

In endometrial cancer, the Wnt/β-catenin signaling pathway is crucial, chiefly due to its role in controlling cell proliferation, differentiation, and survival, factors that are key to tumor development and metastatic progression. Often, the disruption of this pathway correlates with more advanced stages of the disease and is linked to a less favorable prognosis. This understanding is supported by various studies and research in the field of oncology, particularly in the context of solid tumors, including endometrial cancer(Zhang and Wang 2020). It was noted that the upregulation of SOX17 in cells markedly elevated the SOX17 levels (Fig. 5A). In the plate cloning experiment, cells overexpressing SOX17 demonstrated a significantly enhanced proliferative capacity (Fig. 5B). Enhanced cell invasion and migration were observed in the Transwell assay, where cells overexpressing SOX17 showed increased invasiveness and migration ability (Fig. 5C). Increased expression levels of CD31 and VEGFA were noted: further Western Blot analysis indicated that after overexpression of SOX17, the protein expression levels of the endothelial cell marker CD31 and the angiogenic factor VEGFA significantly increased (Fig. 5D). An increase in angiogenic capability was observed in the Tube formation assay, where a significant increase in the number of blood vessels formed was noted in cells overexpressing SOX17 (Fig. 5E). These findings suggest that SOX17, through its activation of the wnt/beta-catenin pathway in endometrial cancer cells, contributes to malignant biological activities like proliferation, invasion, and migration, and also markedly augments angiogenesis.

### Enhanced expression of SOX17 considerably diminishes the suppressive impact of oe-ZBTB7A on malignant biological activities in endometrial cancer cells

**Fig. 6:**
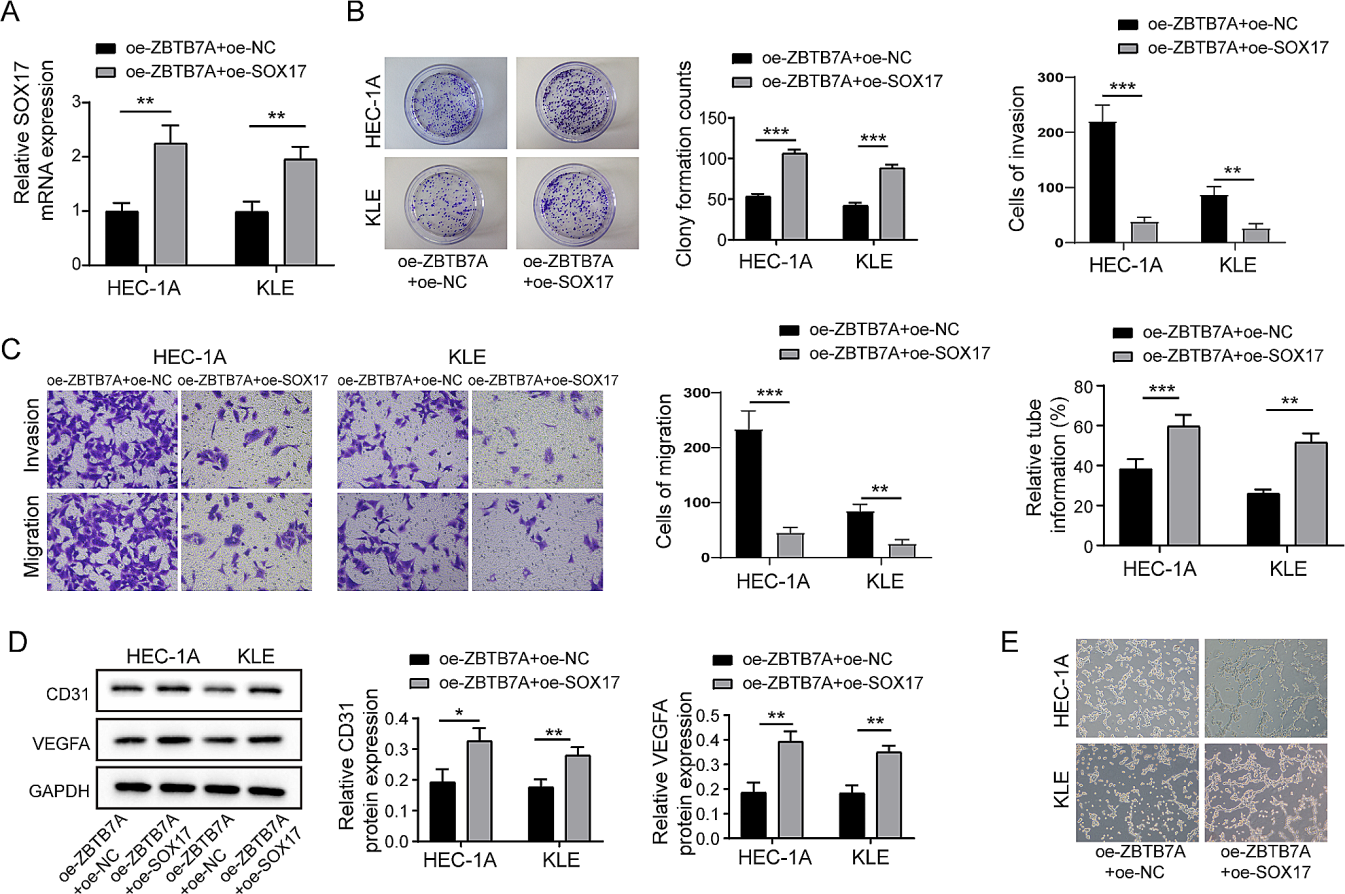
Overexpression of SOX17 Significantly Weakens the Inhibitory Effect of oe-ZBTB7A on Malignant Biological Behaviors in Endometrial Cancer Cells. After further overexpression of SOX17 in HEC-1 A and KLE cell lines with overexpressed ZBTB7A (**A**) There was a significant increase in the levels of SOX17. (**B**) Plate cloning experiments indicated a partial recovery in cell proliferation capability. (**C**) Transwell assays demonstrated a restoration in cell invasion and migration capabilities following SOX17 overexpression. (**D**) Western Blot analysis revealed significant increases in the protein expression levels of CD31 and VEGFA. (**E**) Tube formation assays showed an increase in the number of blood vessels formed. Data from at least three replicates were presented as mean ± standard deviation (SD). *P < 0.05, **P < 0.01

Our subsequent investigation focused on the impact of SOX17 overexpression on the restraining effect of ZBTB7A on malignant biological behaviors in HEC-1 A and KLE endometrial cancer cell lines. It was observed that additional upregulation of SOX17 in these cells resulted in a notable elevation of SOX17 levels (Fig. 6A). The Western Blot analysis revealed a significant elevation in the protein levels of Wnt-1 and β-catenin, suggesting an activation of the wnt/β-catenin pathway (Fig. 6B). Plate cloning experiments demonstrated a partial recovery in cell proliferation (Fig. 6C). Transwell assays revealed that overexpression of SOX17 restored cell invasion and migration capabilities (Fig. 6D); Tube formation assay showed an increase in blood vessel formation (Fig. 6E). To conclude, the findings imply that the enhanced expression of SOX17 considerably reduces the suppressive impact of ZBTB7A on malignant biological activities in endometrial cancer cells.

### ZBTB7A overexpression can drastically suppress cell proliferation, metastasis, and angiogenesis in vivo

**Fig. 7:**
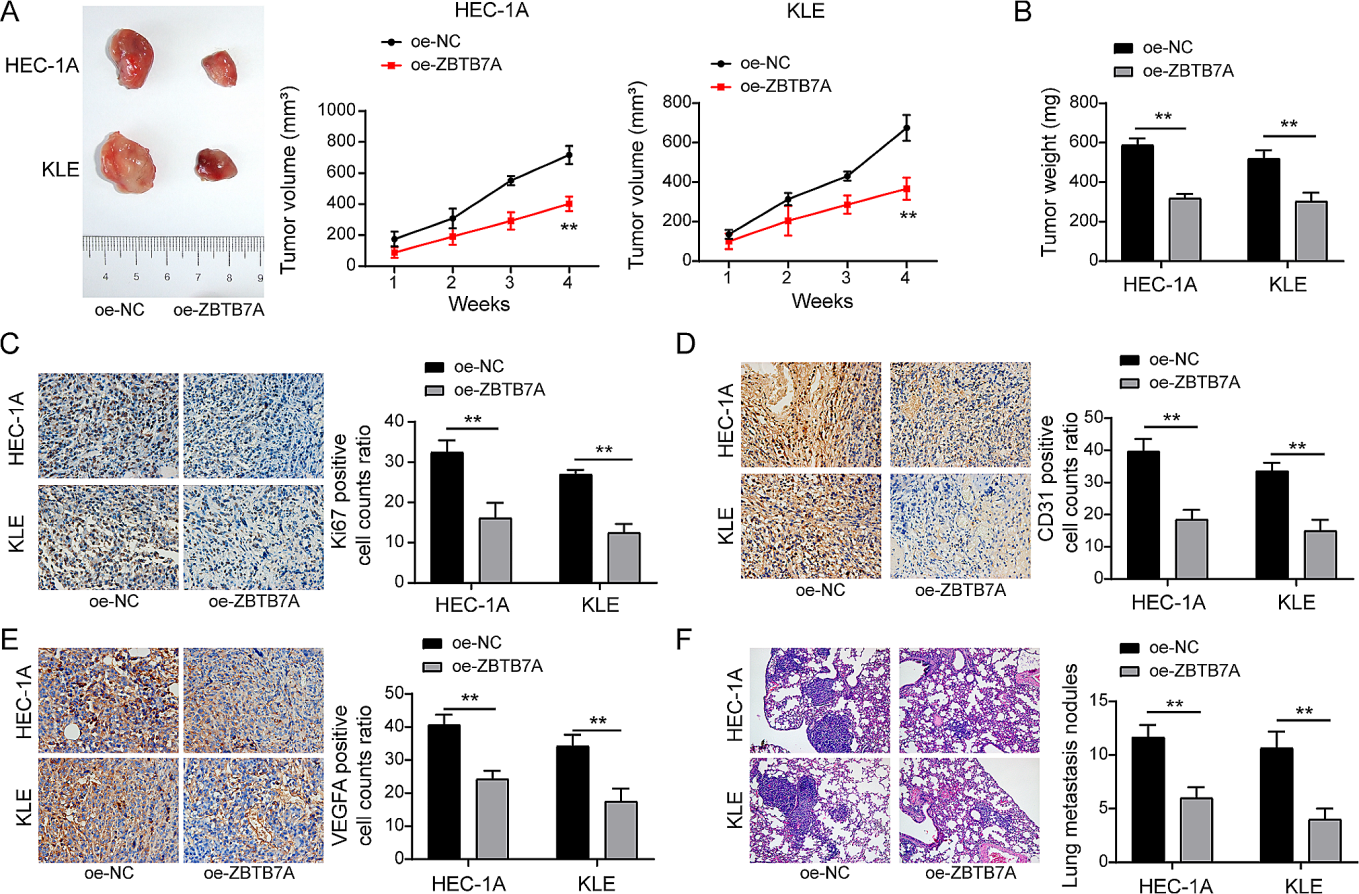
ZBTB7A overexpression dramatically reduced the angiogenesis, metastasis, and proliferation of EC cells in vivo (**A** and **B**) In vivo overexpression of pcDNA-ZBTB7A in HEC-1 A and KLE cells resulted in considerably lower tumour weight and volume compared to the sh-NC-transfected control group. (**C**, **D**, and **E**) The frequencies of cells that were positive for Ki67, CD31, and VEGFA significantly decreased, according to immunohistochemistry. (**F**) HE staining revealed that there were considerably less HEC-1 A and KLE cell-produced nodules in mouse lung tissue. Data from at least three replicates were presented as mean ± standard deviation (SD). *P < 0.05, **P < 0.01

To further support ZBTB7A’s involvement in tumour development, metastasis, and angiogenesis. To assess tumour development, metastasis, and angiogenesis in ZBTB7A and control groups, we built xenograft mice models and tumour metastatic models. The outcomes demonstrated a substantial inhibition of tumour volume and weight in the oe-ZBTB7A group as compared to the oe-NC group (Fig. 7A and B). We also detected the expression of KI-67, CD31, and VEGFA using IHC staining in tumour masses in order to assess the degree of tumour angiogenesis and neoplastic malignancy. According to the findings, oe-ZBTB7A cell-derived tumour masses had much lower positive rates for KI-67, CD31, and VEGFA than oe-NC-derived tumours (Fig. 7C-E). Importantly, HE staining revealed that there were much fewer nodules generated by HEC-1 A and KLE cells in mouse lung tissue (Fig. 7F). In conclusion, these findings showed that ZBTB7A overexpression inhibited EC development, metastasis, and angiogenesis in vivo.

## Discussion

Endometrial cancer is the sixth most common cause of cancer-related illness in women. Despite that removal surgery is the primary and efficient way to treat early-stage EC, the treatment of high-risk, advanced EC remains unsatisfactory (Liu et al. 2019). Growing pieces of evidence showed that lncRNAs are significantly involved in EC progress and the malignant biological behavior of EC cells. LncRNA HOTAIR comes from the transcription of the antisense strand of the HoxC gene and plays important role in chromatin remodeling by recruiting PRC2 to catalyze H3K27me3. Recent studies showed that HOTAIR is significantly involved in EC by regulating EC cell proliferation, migration, EMT, and drug resistance (Zhang et al. 2019). However, the underlying mechanism has not been adequately illustrated. Based on the distinct pathological and histological behavior, EC can be divided into two subtypes, type I and type II. Type I endometrial cancer typically denotes early-stage, low-grade malignancy, characterized by elevated levels of estrogen and progesterone receptors and minimal histological differentiation. The type I EC accounts for the majority of the EC but has a relatively good prognosis. Type II EC usually represents advanced-stage and highly aggressive cancer which is hormone-receptor negative, and have poor survival rates (Nyen et al. 2018; Kozak et al. 2018). Based on different cellular and molecular behavior, the RL95-2 cell line has been considered as a model for type I EC, while HEC-1 A, HEC-1B and KLE cell lines are considered as type II EC-derived cell lines (Kozak et al. 2018; Seleci et al. 2017). Our research revealed an overexpression of lncRNA HOTAIR in both endometrial cancer tissues and cell lines, which correlated with a less favorable prognosis in patients with endometrial cancer. (Fig. 1). Interestingly, we found HOTAIR expressed more in type II EC-derived cell lines (HEC-1 A, HEC-1B and KLE) compared with type I EC-derived cell line RL95-2 (Fig. 1C), suggesting HOTAIR is related to a highly aggressive and invasive phenotype of EC. We knocked down HOTAIR in type II EC-derived cell lines HEC-1 A and KLE to further examine its function in malignant biological activity of EC. We found knockdown of HOTAIR not only impaired both EC cell growth (Fig. 2. A) and angiogenesis (Fig. 1B, C), but also induced EC cell apoptosis (Fig. 2D, E). These results suggest HOTAIR positively regulates the malignant biological behavior of EC cells. Our findings support a prior study that demonstrated HOTAIR mediates estrogen-induced EC cell metastasis (Zhou et al. 2018). . However, our results further proved that HOTAIR plays a broader significance in EC progress as it not only promotes estrogen receptor-positive EC cells but also has a significant effect on estrogen receptor-negative EC cells (KLE).

ZBTB7A, a versatile transcription factor, plays a vital role in cell proliferation and differentiation. Recent studies indicate its role as an oncogenic driver linked to cancer advancement and metastatic spread (Gupta et al. 2020; Singh et al. 2021). A recent study found that ZBTB7A can impede the proliferation and migration of endometrial cancer cells and is positively associated with a more favorable prognosis for endometrial cancer (Khorsandi et al. 2020). The tumor-suppressive mechanism of ZBTB7A is not clear and the relationship of ZBTB7A to HOTAIR has never been explored. We first discovered that ZBTB7A bound to the E3 promoter region of HOTAIR (Fig. 3A-C) and significantly inhibited HOTAIR expression in EC cells (Fig. 3E). Enhanced expression of ZBTB7A markedly suppressed the growth, metastasis, and angiogenesis of endometrial cancer cells in both tumor metastasis and xenograft models in vivo (Fig. 7). These data collectively showed for the first time that ZBTB7A inhibits EC malignancy likely through regulating HOTAIR expression.

Research has shown that SOX17 enables immune evasion in early colorectal adenomas and cancers by suppressing interferon-gamma (IFNγ) signaling, thereby reducing the immunogenicity of tumor cells. This suppression helps colorectal cancer cells evade detection and destruction by the immune system, creating an immunosuppressive microenvironment that facilitates tumor growth and progression Additionally, SOX17 downregulates the expression of major histocompatibility complex class I (MHC-I) molecules, further aiding in immune evasion (Goto et al. 2024a, b; Grimm et al. 2020). . In ovarian cancer, the transcription factor PAX8 has been found to interact with SOX17 to promote angiogenesis, a crucial process for tumor growth and metastasis. This interaction underscores the role of SOX17 in modulating the tumor microenvironment by enhancing blood vessel formation, which supplies the necessary nutrients and oxygen for tumor survival and expansion (Goto et al. 2024a, b). Furthermore, members of the SOX family, including SOX17, are known to play pivotal roles in solid tumors and metastasis. SOX17’s involvement in various signaling pathways and its regulatory functions contribute to changes in the tumor microenvironment that support cancer cell proliferation and metastatic potential (Goto et al. 2024a, b). Overall, these studies indicate that SOX17 is not only crucial for immune evasion but also significantly impacts the tumor microenvironment by promoting angiogenesis and altering the immune landscape, making it a potential target for therapeutic intervention in cancer treatment. Our study has elucidated a crucial pathway in the pathogenesis of endometrial cancer (EC) involving the interaction between ELAVL1 and HOTAIR, which in turn influences the regulation of SOX17. We have demonstrated that this interaction promotes the transcription and functional activity of SOX17, a key regulator in EC. The significant upregulation of SOX17 observed in our findings highlights its potential role in promoting malignant biological behaviors and angiogenesis in EC. This underscores SOX17 as a critical factor in the development and progression of endometrial cancer, potentially serving as a target for therapeutic intervention.

The elucidation of the ELAVL1-SOX17 axis offers new avenues for targeted therapy in endometrial cancer (EC). Given the pivotal role this axis plays in EC progression, targeting either ELAVL1 or SOX17 could effectively disrupt this oncogenic network. Potential therapeutic strategies could include small molecule inhibitors, RNA-based therapies, or antibody-based treatments aimed at inhibiting key components or interactions within this axis. The ability to specifically target this pathway could lead to more effective treatments with fewer side effects compared to conventional therapies, thereby improving patient outcomes.

## Data Availability

All data generated or analysed during this study are included in this article.

## Abbreviations

EC: Endometrial carcinoma
ZBTB7A: Zinc Finger And BTB Domain Containing 7A
lncRNA: Long non-coding RNA
sh-RNA: Short hairpin RNASOX17: SRY-Box Transcription Factor 17
ELAVL1: ELAV Like RNA Binding Protein 1
HOTAIR: Hox transcript antisense intergenic RNA
H3K27me3: Trimethylation of Lys-27 in histone 3
RT-qPCR: Quantitative reverse-transcription polymerase chain reaction
TBST: Tris buffered saline Tween
PVDF: Polyvinylidene difluoride
ATCC: American Type Culture Collection
DMEM: American Type Culture Collection
ATCC: Dulbecco?s Modified Eagle?s Medium
FBS: Fetal bovine serum
HOTAIR HOX: Transeript antisense intergenitic RNA
HE: Hematoxylin-eosin
lncRNAs: Long non-coding RNAs
si-NC: Negative control siRNA
PI3K/Akt: Phosphatidylinositol-3-kinase/protein kinase B
PVDF: Polyvinylidene difluoride membrane
RIPA: Radio-Immunoprecipitation Assay
SDS-PAGE: Sodium dodecyl sulfate?polyacrylamide gel electrophoresis
SD: Standard deviation
TUNEL: Terminal deoxynucleotidyl transferase-mediated dUTP-biotin nick end labeling
